# PAK4 Kinase Activity Plays a Crucial Role in the Podosome Ring of Myeloid Cells

**DOI:** 10.1016/j.celrep.2019.11.016

**Published:** 2019-12-10

**Authors:** Elizabeth Foxall, Adela Staszowska, Liisa M. Hirvonen, Mirella Georgouli, Mariacristina Ciccioli, Alexander Rimmer, Lynn Williams, Yolanda Calle, Victoria Sanz Moreno, Susan Cox, Gareth E. Jones, Claire M. Wells

**Affiliations:** 1 Randall Centre for Cell and Molecular Biophysics, King’s College London, London, UK; 2 Department of Life Sciences, University of Roehampton, London, UK; 3 School of Cancer and Pharmaceutical Sciences, King’s College London, London, UK; 4 Barts Cancer Institute, Queen Mary University of London, London, UK; 5 Kennedy Institute of Rheumatology, Oxford University, Oxford, UK

**Keywords:** PAK4, podosome, adhesion, migration, superresolution microscopy, AKT

## Abstract

p21-Activated kinase 4 (PAK4), a serine/threonine kinase, is purported to localize to podosomes: transient adhesive structures that degrade the extracellular matrix to facilitate rapid myeloid cell migration. We find that treatment of transforming growth factor β (TGF-β)-differentiated monocytic (THP-1) cells with a PAK4-targeted inhibitor significantly reduces podosome formation and induces the formation of focal adhesions. This switch in adhesions confers a diminution of matrix degradation and reduced cell migration. Furthermore, reduced PAK4 expression causes a significant reduction in podosome number that cannot be rescued by kinase-dead PAK4, supporting a kinase-dependent role. Concomitant with PAK4 depletion, phosphorylation of Akt is perturbed, whereas a specific phospho-Akt signal is detected within the podosomes. Using superresolution analysis, we find that PAK4 specifically localizes in the podosome ring, nearer to the actin core than other ring proteins. We propose PAK4 kinase activity intersects with the Akt pathway at the podosome ring:core interface to drive regulation of macrophage podosome turnover.

## Highlights

• PAK4 kinase activity is crucial to macrophage podosome dynamics
• Podosome loss due to PAK4 suppression reduces migration speed and degradation
• PAK4 localizes to the podosome ring where it interacts with vinculin and paxillin

## Graphical Abstract

**Figure:**
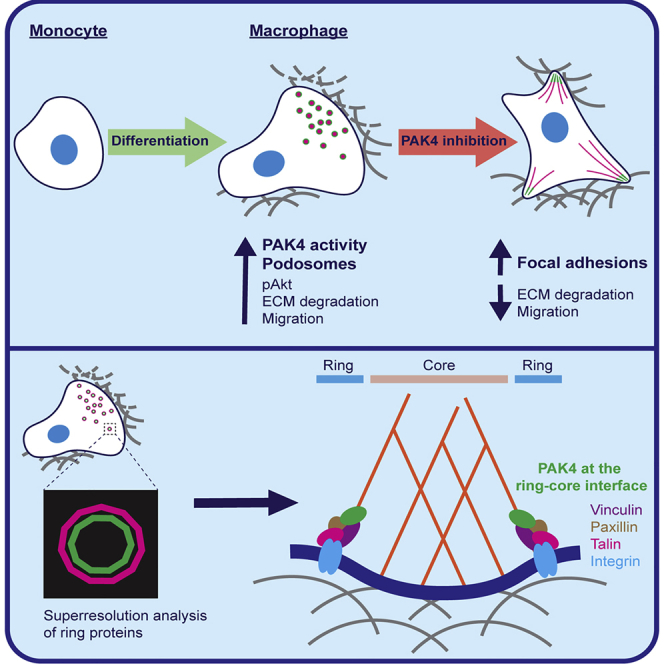

## Teaser

The differential role of PAK family proteins in podosome function has not been previously evaluated. Foxall et al. demonstrate that PAK4 rather than PAK1 plays a pivotal role and use super resolution microscopy to position PAK4 at the podosome ring:core interface.

## Introduction

Previously, PAK4 was found to partially colocalize with podosome F-actin cores in primary macrophages, and short hairpin RNA (shRNA)-mediated knockdown of PAK4 reduced podosome numbers (Gringel et al., 2006). In this model, cells expressing the kinase-dead mutant PAK4 (K350M) experienced a small decrease in podosome numbers and reduced podosome size. In contrast, cells expressing the constitutively active mutant PAK4 (S445N,S474E) did not significantly elevate podosome numbers but did exhibit an increased podosome size (Gringel et al., 2006). As the cells in this study retained expression of endogenous PAK4, this may account for the modest changes in podosome numbers observed. Moreover, it is not clear if PAK4Δkinase localizes to the podosome; thus, the involvement of PAK4 kinase activity in podosome turnover is still an open question. Recently, PAK4 kinase activity was shown to play an essential role in the maturation of invadopodia—a cell adhesion structure related to podosomes (Nicholas et al., 2016). Moreover, a second PAK family member (PAK1) was also implicated in regulating invadopodial turnover. Currently, the role of PAK1/PAK2 in podosomes remains unclear. Exogenous expression of PAK1 and PAK2 were shown to decrease podosome number in src-transformed fibroblasts by phosphorylation of caldesmon (Morita et al., 2007); in contrast, expression of PAK1 in smooth muscle cells and its interaction with PIX increased podosome-like F-actin puncta (Nayal et al., 2006, Webb et al., 2005).

## Results and Discussion

### PAK4 Kinase Activity Drives Podosome Formation

**Figure 1:**
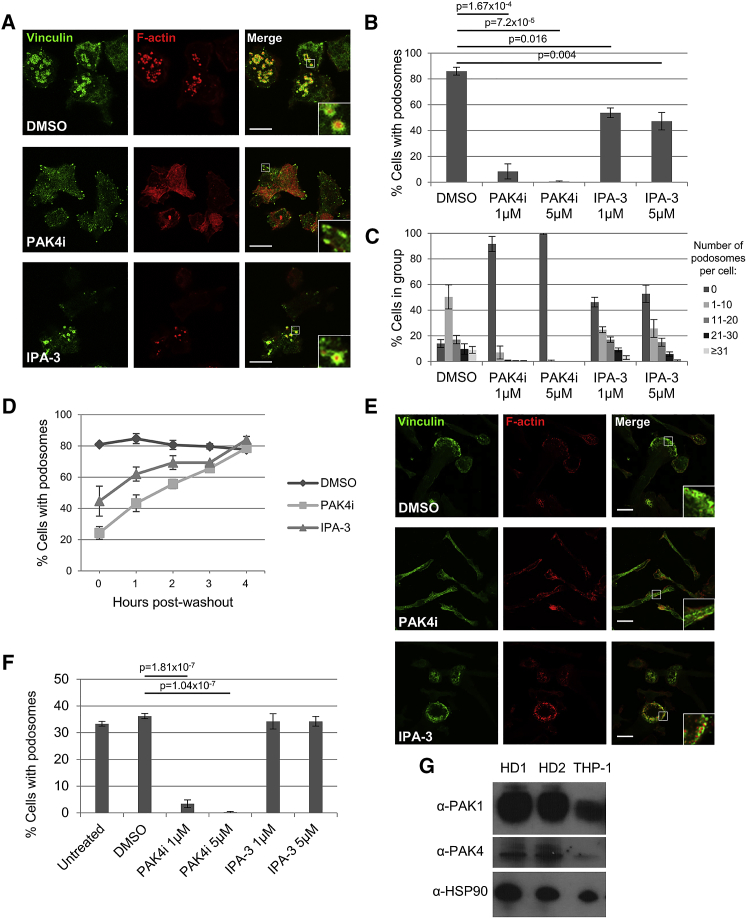
PAK4 Kinase Activity Drives Podosome Formation (A) Confocal images of THP-1 cells seeded on fibronectin with TGF-β for 16 h and then treated for 4 h with DMSO control or 1 μM PAK inhibitors PAK4i or IPA-3. Stained for vinculin (green) and F-actin (red). Insert zoom of podosomes or peripheral adhesions. (B and C) Percentage of THP-1 cells with podosomes following treatment with DMSO or 1 or 5 μM PAK inhibitors for 4 h (B) and the percentage of cells with 0, 1–10, 11–20, 21–30, or ≥31 podosomes per cell was calculated from 300 cells per condition (C). (D) Following a 4 h treatment with DMSO or inhibitors, the inhibitors were washed out and cells incubated with TGF-β for a further 4 h. Cells were fixed and stained for vinculin and F-actin at times indicated, and the percentage of cells with podosomes were counted. (E) Primary human monocytes isolated from peripheral human blood from healthy donors were seeded on fibronectin and differentiated toward macrophages by incubating for 4.5 days with 50 ng/ml M-CSF. Macrophages were then treated with DMSO or 1 μM PAK inhibitor PAK4i or IPA-3, fixed, and stained for vinculin (green) and F-actin (red). (F) Percentage of primary human macrophages with podosomes following a 4 h treatment with DMSO or 1 or 5μM PAK inhibitors. (G) Western blot for PAK1 and PAK4 levels in lysates of primary monocytes from two healthy donors (HD1 and HD2) cultured for 6 days, alongside a THP-1 lysate from cells differentiated for 16 h with TGF-β. For all graphs, error bars represent ±SEM, and p values indicate significant difference to DMSO-treated cells by one-way ANOVA. Scale bars in (A) and (E) represent 10 μm.

Given that the function of PAK1 and PAK4 kinase activity in podosomes remains unclear (Gringel et al., 2006) and that PAK1 and PAK4 show distinct roles in invadopodia dynamics (Nicholas et al., 2016), we decided to exploit a recently developed PAK4-targeted small-molecule ATP-competitive inhibitor (Whale et al., 2013) (herein called PAK4i; see Key Resources Table for details) and a PAK1-specific inhibitor (IPA-3) (Viaud and Peterson, 2009) to elucidate the requirement for PAK1/PAK4 kinase activity in podosome turnover. To this aim, we have used the THP-1 human monocyte cell line, where stimulation of cells with transforming growth factor β (TGF-β) promotes the formation of podosomes (Bombara and Ignotz, 1992, Rafiq et al., 2017, Zhang et al., 2016). Initially, we confirmed that these cells express both PAK1 and PAK4 (Figure S1A). To monitor podosome formation, THP-1 cells were seeded on fibronectin and stimulated with TGF-β. Cells were then incubated with DMSO vehicle control, PAK4i, or IPA-3. Cells were stained for F-actin to localize podosome cores and vinculin to highlight the podosome ring (Vijayakumar et al., 2015). Although incubation with DMSO had no impact on podosome formation (Figures S1B–S1D), incubation with either PAK4i or IPA-3 significantly inhibited the formation of TGF-β-induced podosomes (Figures 1A and 1B). Moreover, kinase inhibition also suppresses the number of podosomes per cell (Figure 1C). This is in agreement with previous work demonstrating that expression of kinase-dead PAK4 suppresses the number of podosomes per cell (Gringel et al., 2006). Importantly, removal of the inhibitors allowed the cells to recover podosomes (Figure 1D), demonstrating that inhibition is not toxic and does not irreversibly block the formation of podosomes. These data suggest that PAK1 and PAK4 both play a role in podosome formation; however, we observed a consistently greater loss of podosomes in the presence of PAK4i compared to IPA-3 (Figure 1B). THP-1 cells are a well-established model system to study podosomes (Bombara and Ignotz, 1992, Rafiq et al., 2017, Zhang et al., 2016); however, we felt it important to test our findings in a primary setting. We thus isolated primary peripheral blood mononuclear cells (PBMCs) from two human donors and differentiated these cells toward macrophages by culture in macrophage colony stimulating factor (M-CSF) (Lacey et al., 2012, Martinez et al., 2008). Interestingly, incubation with IPA-3 did not inhibit podosome formation (Figures 1E and 1F; Figure S1E). In contrast, incubation with PAK4i almost entirely suppressed podosome formation in these cells. The lack of IPA-3 impact is not due to a low level of PAK1 expression, as there is a readily detectable level of PAK1 in these cells (Figure 1G). Previous work suggested that expression of PAK4Δkinase enhanced the level of podosome formation (Gringel et al., 2006), although it was not localized to the podosome. We would speculate that additional/*de novo*-binding interactions of PAK4Δkinase precipitated podosome formation in a background of endogenous PAK4 activity. Our data now clarify the importance of PAK4 kinase activity for podosome formation.

### Loss of PAK-Dependent Podosome Formation Impairs Migration

**Figure 2:**
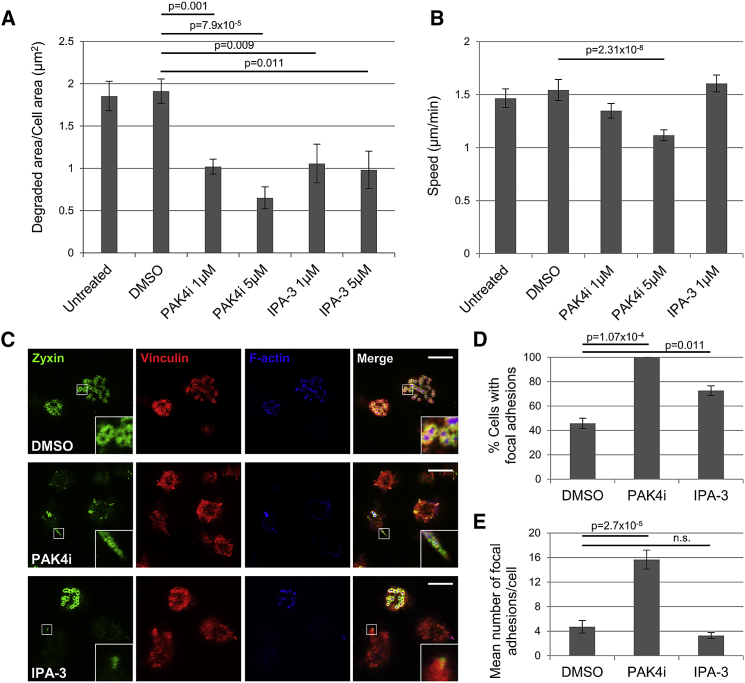
Podosome Loss Following PAK Inhibition Is Accompanied by an Increase in Focal Adhesions and a Reduction of Invasive Migration (A) For the matrix degradation assay, >20 fields of view per treatment condition were measured. (B) Mean cell speed (μm/minute) was calculated from >90 cells from three separate experiments. (C) Confocal images of THP-1 cells seeded on fibronectin with TGF-β for 16 h and then treated for 4 h with 1 μM PAK inhibitors. Cells were stained for zyxin (green), vinculin (red), and F-actin (blue). Insert zoom of peripheral adhesion. Scale bars represent 10 μm. (D) Percentage of cells with focal adhesions. (E) Number of focal adhesions/cell. For all graphs, error bars represent ±SEM, and p values denote significant difference to DMSO-treated cells by one-way ANOVA.

Macrophages require mature, degradative podosomes to migrate efficiently (Burns et al., 2004, Calle et al., 2008, Cougoule et al., 2010, Dehring et al., 2011, Ley et al., 2007, Olivier et al., 2006). Using a matrix degradation assay, we next tested if podosome loss as a result of PAK inhibition leads to a loss of matrix degradation in differentiated THP-1 cells. Incubation with DMSO did not significantly inhibit fibronectin degradation (Figure 2A). In contrast, incubation with IPA-3 and PAK4i significantly inhibited matrix degradation, although, as previously observed, PAK4i had a greater impact (Figure 2A; Figure S2A). Thus, incubation with PAK inhibitors suppresses macrophage functionality. The impact of IPA-3 on matrix degradation suggests that there is a specific disconnect in the MMP (Matrix metalloproteinase) delivery pathway. Disruption of a PAK1-cortactin interaction causes some cancer cells to exhibit stabilized invadopodia but reduced ability to invade (Jeannot et al., 2017), suggesting a specific role for PAK1 in matrix degradation. Moreover, cortactin is required for secretion of MMP1 at podosome sites to mediate degradation (Bañón-Rodríguez et al., 2011, Clark et al., 2007). To complete our studies, we next tested the ability of PAK-inhibited cells to efficiently migrate (Figure 2B; Figure S2B). Interestingly, only inhibition of PAK4 impeded cell migration speed at higher concentrations (Figure 2B). Thus, the remaining podosomes in IPA-3-treated cells (Figure 1B) are sufficient to promote migration; however, treatment of cells with 5 μM IPA-3 induced significant cell detachment in this assay and so cell migration potential could not be evaluated (Figure S2C). The substantial loss of podosomes in PAK4i-treated cells delivers a significant impact on cell migration speed, again suggesting that PAK4 plays a more prominent role than PAK1 in podosome turnover and cell migration. It should be noted that podosomes are not always intrinsically linked to cell migration potential. In myeloid cells that are depleted of WASP expression, there are no podosomes and their migration is badly affected (Burns et al., 2001, Linder et al., 1999) but not intrinsically blocked (Binks et al., 1998). Indeed, cells treated with 1 μM PAK4i have a significant reduction in podosome number but retain migration potential. This of course may be accounted for by the induction of focal adhesions in these cells. It would be interesting to test the link between podosome formation and 3D invasion (Cougoule et al., 2010, Cougoule et al., 2018). Nevertheless, in our studies inhibition of PAK4 kinase activity clearly impacts both podosome formation and cell migration.

### Adhesion Switching Occurs in PAK4i-Treated Cells

In addition to their roles in invadosome dynamics, both PAK1 and PAK4 have been implicated in the regulation of focal adhesions (Dart et al., 2015, Nayal et al., 2006, Zhao et al., 2000). Thus, we made a detailed analysis of adhesion formation in treated cells. Cells were co-stained for vinculin to localize podosomes and zyxin to localize mature focal adhesions (Block et al., 2008, Nobes and Hall, 1995, Zamir and Geiger, 2001). Interestingly, we detected a differential response in the PAK4i- versus IPA-3-treated cells (Figures 2C–2E). Cells incubated with DMSO and IPA-3 exhibited similar numbers of focal adhesions per cell (Figure 2E), although IPA-3 treatment did increase the number of cells with focal adhesions (Figure 2D). In contrast, treatment of cells with PAK4i led to a dramatic increase in the number of cells with focal adhesions and the number of focal adhesions per cell. This observation has been previously described for PAK4-depleted cells (Dart et al., 2015, Wells and Jones, 2010), although this was not associated with kinase activity. Indeed, the increase in focal adhesions can also be observed in differentiated PBMCs (Figure 1E; Figure S1E). Furthermore, treatment with PAK4i did not reduce cell-matrix adhesion capacity, whereas incubation with IPA-3 significantly reduced cell adhesion to the extent that treatment with 5 μm IPA-3 led to undetectable cell numbers attached to the substratum (Figure S2C). Thus, it is likely that the increase in focal adhesions observed in PAK4i-treated cells, in part, accounts for the reduced migration speed (Wells and Jones, 2010). Whether the formation of focal adhesions is a direct consequence of reduced PAK4 activity remains unclear; it is widely reported that when cells are no longer able to make podosomes they switch to making peripheral adhesions. Therefore, the increase in peripheral adhesions in PAK4i-treated THP-1 cells is likely an indirect effect, as PAK4 function in peripheral adhesion turnover is kinase independent (Dart et al., 2015). In contrast, PAK4 kinase activity is intrinsically linked to the maturation of invadopodia where it is required for the suppression of RhoA activity (Nicholas et al., 2016). Interestingly, low RhoA activity was observed in fibroblasts forming podosomes when plated on a soft matrix (Bays et al., 2014), whereas activation of RhoA was found to result in podosome dissolution (Rafiq et al., 2017). We, therefore, suggest that PAK4 may act to suppress RhoA activity in podosomes, and in the absence of this suppression increased RhoA activity promotes the formation of focal adhesions (Rafiq et al., 2017). In our studies, IPA3-treated cells lose some podosomes but are unable to promote focal adhesion formation (Figures 2C–2E and S2C); this suggests a differential role for PAK1. Indeed, PAK1 has previously been associated with focal adhesion formation (Nayal et al., 2006). Overall, our data point to a substantial role for PAK4 in macrophage podosomes; we therefore decided to focus on PAK4 for extended studies.

### PAK4 Depletion Suppresses Podosome Turnover in a Kinase-Dependent Manner

**Figure 3:**
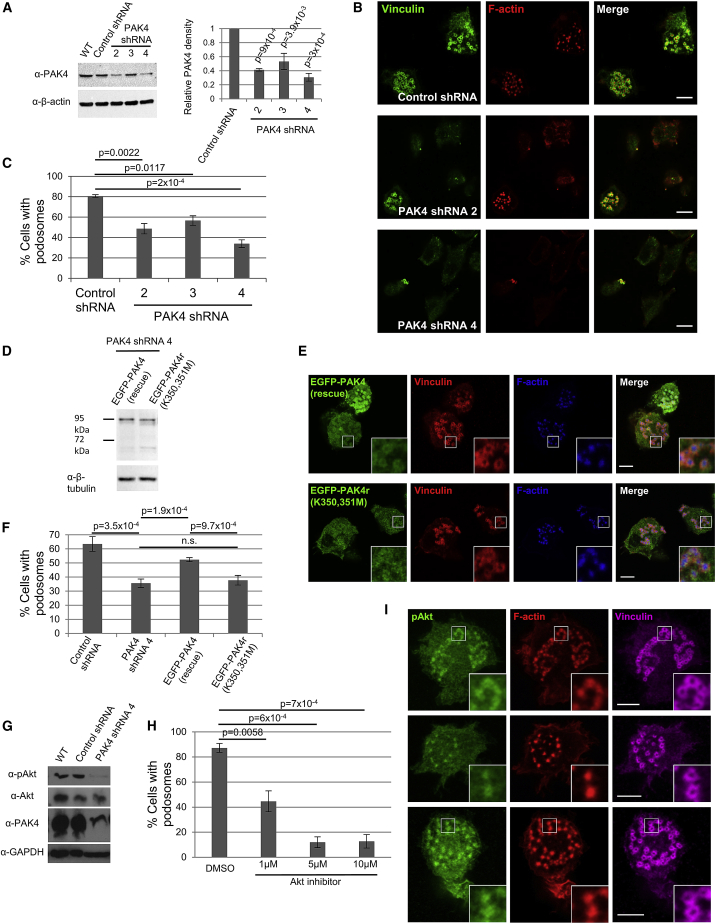
PAK4 Knockdown and Rescue Supports a Kinase-Dependent Role for PAK4 in Podosomes through the Activation of Akt (A) PAK4 shRNAs or scrambled control cells were probed for PAK4 expression. (B) PAK4 shRNA-expressing cells were seeded on fibronectin with TGF-β for 16 h, fixed, and stained for vinculin (green) and F-actin (red). (C) Percentage of cells with podosomes (>300 cells per cell line). (D) PAK4 knockdown cells (A) expressing EGFP-tagged shRNA-resistant PAK4: EGFP-PAK4 (rescue) or the kinase dead mutant PAK4: EGFP-PAK4r (K350,351M) were probed for PAK4 expression. (E) Confocal images of PAK4 rescue THP-1 cells. (F) Percentage of cells with podosomes. (G) Cells were probed for pAkt and PAK4. (H) THP-1 cells on fibronectin with TGF-β for 16 h were treated with indicated concentrations of Akt inhibitor for 4 h, fixed and stained for F-actin, and scored for podosomes. (I) Confocal images of THP-1 cells fixed and stained for pAkt, F-actin, and vinculin. Error bars = ± SEM and p values indicate significant differences between treated cells by one-way ANOVA. Scale bars in (B) and (E) = 10 μm; scale bars in (I) = 5 μm.

Because PAK4i may also affect PAK5/PAK6 (likely to be minimal—see Key Resources Table), we generated stable PAK4 knockdown cell lines by using three different shRNA sequences (Figure 3A) to complement our inhibitor studies and negate any off-target effects. Cells stably depleted of PAK4 expression did not modulate expression of the other ubiquitously expressed PAKs, namely, PAK1 and PAK6 (Figure S3A). THP-1 cells expressing a control shRNA sequence were able to efficiently generate podosomes in the presence of TGF-β, whereas cells depleted of PAK4 expression were unable to generate podosomes to control levels (Figures 3B, 3C, and S3B). PAK4 knockdown cells also display peripheral focal adhesions (Figure 3B), although this phenotype is less pronounced than in PAK4i-treated cells; this is likely due to PAK4 knockdown cells retaining low PAK4 expression and low numbers of podosomes. Importantly, we aimed to confirm the requirement for kinase activity. We generated two rescue cell lines (Figure 3D): PAK4shRNA4 expressing EGFP-PAK4 (rescue) and PAK4shRNA4 expressing an siRNA-resistant, kinase-dead variant EGFP-PAK4r (K350,351M) (Wells et al., 2002). Re-expression of wild-type PAK4 was able to significantly increase the number of podosome-positive cells (Figures 3E, 3F, and S3C). Re-expression of wild-type PAK4 in PAK4shRNA4 cells did not completely rescue podosomes to control levels. This is a particularly challenging experiment as the level of PAK4 expression needs to be carefully controlled to prevent analysis of PAK4-overexpressing cells where cell rounding is likely to occur (Wells et al., 2002). It is, therefore, likely that some podosome-positive cells were excluded from the analysis based on morphology. However, we achieve over 80% rescue that is consistent with previous PAK4 shRNA migratory rescue experiments (Dart et al., 2015, Whale et al., 2013) and levels of recovery more generally observed when rescuing kinase phenotypes (Rannou et al., 2008, Nalepa et al., 2013). Importantly, re-expression of the kinase-dead variant was unable to deliver any phenotypic rescue (Figures 3E, 3F, and S3C). Taken together with our inhibitor studies, these data suggest that PAK4 plays a prominent and essential role in podosome formation across cell types, including primary human myeloid cells, and that kinase activity at podosomes is critical to PAK4 function.

We and others have recently identified a specific impairment in Akt signaling in PAK4-depleted cells (Jeannot et al., 2017). However, Akt activity has not been previously associated with myeloid cell podosome formation. We found that levels of Akt phosphorylation were suppressed in PAK4sh4RNA-expressing cells (Figure 3G), whereas pcofilin and pLIMK levels were unaffected (Figure S3D). In contrast, treatment with IPA-3 did not modulate pAkt levels (Figure S3E). Moreover, we were able to establish that Akt activity is specifically required for podosome formation. Incubation of differentiated cells with an Akt inhibitor significantly suppressed podosome formation (Figure 3H), concomitant with a suppression of PRAS40 phosphorylation (Figure S3F). Importantly, subsequent removal of Akt inhibition restored podosome levels to control (Figure S3G). Recently, a phosphorylated Akt signal was detected at invadopodia (Sarwar et al., 2019); using the same antibody, we detected phosphorylated Akt within the podosome (Figure 3I). We do not detect a direct interaction between PAK4 and Akt; we would, therefore, suggest that an important role of PAK4 in podosome formation is the activation of the Akt pathway but not by direct phosphorylation of Akt.

### PAK4 Is Localized to the Podosome Ring

**Figure 4:**
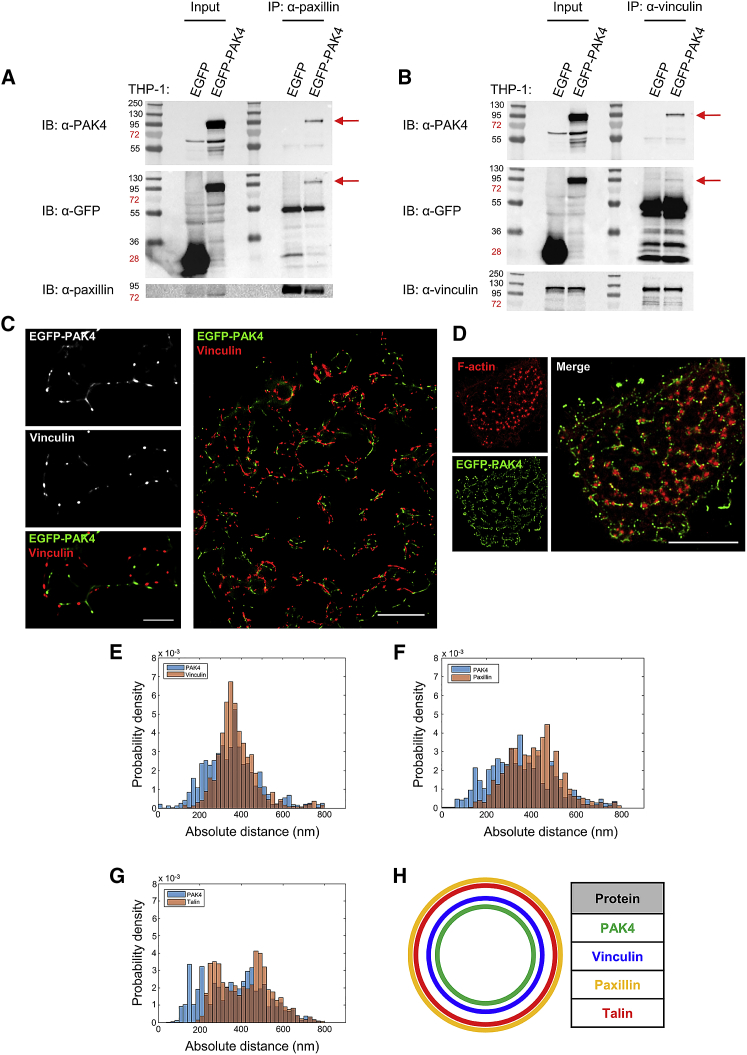
PAK4 Localizes to the Podosome Ring (A and B) THP-1 cells stably expressing EGFP or EGFP-PAK4 were seeded on fibronectin with TGF-β for 16 h prior to immunoprecipitation (IP) of paxillin (A) or vinculin (B). Blots were probed for endogenous PAK4 and reprobed for GFP and paxillin or vinculin. (C) EGFP-PAK4-expressing THP-1 cells were seeded on fibronectin with TGF-β for 16 h, fixed, and stained for vinculin. Datasets of 200 images were taken for both EGFP-PAK4 and vinculin and analyzed using the ImageJ 3B plugin. Left panels are reconstructed localizations from two adjacent podosomes (reconstruction blur FWHM = 20 nm), and these localizations are representative of >50 podosomes analyzed using 3B; scale bar represents 500 nm. Right panel shows a reconstructed dataset from 3B analysis carried out using a computer cluster to give localizations in podosomes of an entire THP-1 cell; scale bar represents 2 μm. (D) STORM and 3B localization of F-actin and EGFP-PAK4, respectively. Scale bar represents 5 μm. (E–G) 3B datasets generated for >50 podosomes from >10 EGFP-PAK4-expressing THP-1 cells stained for vinculin (E), paxillin (F), or co-expressing mCherry-Talin (G) were analyzed using ring analysis software (Staszowska et al., 2017). Histograms show the absolute distances from the podosome center of EGFP-PAK4 (blue) and vinculin/paxillin/mCherry-Talin (red). (H) A top-down representation of the relative localizations of PAK4 and the podosome ring proteins vinculin, paxillin, and talin, based on 3B localizations.

To gain further insight into PAK4’s functional role, we took a high-resolution microscopy approach to pinpoint the area of PAK4 activity within the podosome. Close inspection of GFP-tagged PAK4 expression in rescue cells (Figure 3E) revealed an interesting discovery regarding the localization of PAK4. EGFP-PAK4 was clearly localizing to the podosome ring rather than the podosome core (Figure 3E). This was not an anomaly of expressing the rescue construct, as we were able to detect ring localization in wild-type cells overexpressing EGFP-PAK4 (Figures S4A and S4B). In contrast, kinase-dead PAK4 was less clearly localized (Figure 3E), again supporting a functional role for PAK4 kinase activity. Podosomes are highly ordered structures, and localization to the ring versus core would change the possible interacting partners and functional consequences. Furthermore, our observation places a serine/threonine kinase in the podosome ring. We hypothesized that if PAK4 is indeed a ring protein, PAK4 should co-immunoprecipitate with ring but not core proteins. Crucially, we detected PAK4 in immunoprecipitations of two ring proteins, paxillin and vinculin, from GFP-PAK4-expressing cells (Figures 4A and 4B) and a faint trace of endogenous PAK4 when a large number of cells was used (Figure S4C). In contrast, we did not detect any PAK4 in an anti-WASP immunoprecipitation (a core protein) (Figure S4D). These biochemical studies support our localization imaging but do not provide irrefutable evidence of PAK4 localization.

### Nanoscale Resolution of PAK4 Localization

To robustly query the indicated localization of PAK4 in the podosome ring, we adopted the Bayesian analysis of blinking and bleaching (3B analysis), which is able to reveal podosome organization at the nanoscale (Cox et al., 2011). EGFP-PAK4-expressing THP-1 cells were seeded on fibronectin in the presence of TGF-β, fixed, and stained for vinculin or paxillin (Figures 4C and S4E). Alternatively, EGFP-PAK4/mCherry-Talin-expressing cells were seeded on fibronectin in the presence of TGF-β and fixed (Figure S4F). Datasets of 200 images were taken using stream acquisition for all of the above conditions and processed using the ImageJ 3B plugin or C++ software (Cox et al., 2011, Rosten et al., 2013). Reconstructed localizations from one or two adjacent podosomes clearly illustrate that PAK4, vinculin, paxillin, and talin localize to the podosome ring (Figures 4C, S4E, and S4F). Moreover, reconstructions of entire THP-1 cells clearly illustrate the ring localization across multiple podosomes (Figures 4C, S4E, and S4F). To further validate our findings, we have also localized PAK4 with respect to the actin core (Figure 4D).

Using the reconstructed localizations, we then calculated the distances of each protein from the podosome center in a given segment of the podosome ring, as described in Staszowska et al. (2017). The absolute positions of EGFP-PAK4 localizations were compared to those of vinculin, paxillin, and mCherry-Talin (Figures 4E–4G). To account for podosome size variations, we also calculated the relative positions of these proteins by subtracting the distances of vinculin, paxillin, and mCherry-Talin from EGFP-PAK4 distance; negative values indicate that PAK4 is closer to the podosome center (Figures S4G–S4I). The mean absolute distances from the podosome center were calculated to be 362 nm, 383 nm, 420 nm, and 426 nm for PAK4, vinculin, talin, and paxillin, respectively. Taken together, our studies now reveal that PAK4 is definitely localized to the podosome ring and occupies a volume internal to the other ring proteins analyzed, placing PAK4 closest to the podosome core (Figure 4H). Thus, our studies have demonstrated that PAK4 kinase activity is essential for podosome formation and that PAK4 specifically functions within the podosome ring in monocytic cells.

It remains to be elucidated how PAK4 is being regulated within this process. PAK4 binds preferentially to Cdc42; however, binding to Cdc42 was suggested to be an intermediate step to PAK4 activation, acting to localize PAK4 activity to specific subcellular compartments (Ha et al., 2012). Active Cdc42 localizes to podosomes and promotes their formation (Daubon et al., 2011, Moreau et al., 2003, Tatin et al., 2006); it is generally considered a core protein given its interaction with WASP, a component of the Arp2/3-mediated F-actin core. Thus, binding of PAK4 to Cdc42 might occur at the core-ring interface, as our data put PAK4 closest to the core. Interestingly, it has been proposed that full activation of PAK4 requires a secondary binding to release the inhibitory binding of a pseudosubstrate sequence to the kinase domain (Abo et al., 1998, Ha et al., 2012). The binding of the Src SH3 domain increased PAK4 activity (Ha et al., 2012), and Src is a well-established regulator of podosome dynamics and cell migration (Timpson et al., 2001); therefore, Src may directly promote PAK4 activity in podosomes. Src is often depicted as a ring protein, but this has not been experimentally confirmed; it is thus possible that Src could also reside at the same core-ring interface along with PAK4.

Our data point to the Akt pathway as a potential target for PAK4 activity within the podosome. However, there may be other alternative or additional targets. PAK4 can promote paxillin phosphorylation at focal adhesions (Nayal et al., 2006, Wells and Jones, 2010). Whether PAK4 mediates phosphorylation of paxillin at podosomes is unknown. PAK4 also interacts with vinculin in podosome-forming THP-1 cells and focal adhesion-forming breast cancer cells (Dart et al., 2015). Whether PAK4 can directly phosphorylate vinculin is unknown, and vinculin phosphorylation on Ser/Thr residues has not been demonstrated. However, Src-mediated tyrosine phosphorylation of vinculin was found to regulate its conformation, affecting force transmission at focal adhesions and cell-cell junctions (Auernheimer et al., 2015, Bays et al., 2014, Huang et al., 2014, Ito et al., 1982). It is also possible that a different protein target of PAK4 resides within the podosome ring, perhaps PDZ-RhoGEF given that a reduction in RhoA activity might be required for podosome formation. Thus, the critical downstream target of PAK4 in podosome turnover remains to be elucidated.

In conclusion, we have shown that PAK4 kinase activity is essential in the regulation of macrophage podosomes. Inhibition of PAK4 results in a dramatic loss of podosomes and the formation of focal adhesions, both in differentiated THP-1 cells and dramatically in primary human macrophages. shRNA-mediated knockdown of PAK4 also reduces podosome number; this phenotype cannot be rescued by a kinase-dead variant of PAK4. Importantly, this is a report of serine/threonine kinase activity as a crucial component of podosome turnover. The switch from podosomes to focal adhesions confers a reduction in cell migration speed and matrix degradative ability, demonstrating a crucial role for PAK4 in promoting macrophage migration by the modulation of adhesion phenotype. Finally, we have shown that PAK4 localizes to the podosome ring by superresolution 3B analysis and that PAK4 is closer to the podosome core than vinculin, paxillin, and talin.

## STAR★Methods

### Key Resources Table

**Table:** 

REAGENT or RESOURCE	SOURCE	IDENTIFIER
**Antibodies**		

Rabbit polyclonal anti-PAK4	In-house (Wells et al., 2002)	N/A
Rabbit polyclonal anti-PAK4	Cell Signaling Technology	Cat. # 3242; RRID:AB_2158622
Rabbit polyclonal anti-PAK1	Cell Signaling Technology	Cat. # 2602; RRID:AB_330222
Rabbit polyclonal anti-PAK1	Santa Cruz Biotechnology	Cat. # sc-882; RRID:AB_672249
Rabbit polyclonal anti-PAK2	Cell Signaling Technology	Cat. # 2608; RRID:AB_2283388
Rabbit polyclonal anti-PAK3	Cell Signaling Technology	Cat. # 2609; RRID:AB_2225298
anti-PAK6	GeneTex	Cat # GTX127915; RRID:AB_2687660
Mouse monoclonal anti-GFP	Roche	Cat. # 11 814 460 001; RRID:AB_390913
Mouse monoclonal anti-vinculin	Sigma	Cat. # V9131-.2ML; RRID:AB_477629
Rabbit polyclonal anti-paxillin	Novus Biologicals	Cat. # NBP1-19833; RRID:AB_1642794
Rabbit polyclonal anti-zyxin	Invitrogen	Cat. # PA1-25162; RRID:AB_2221183
Mouse monoclonal anti-WASP	Santa Cruz Biotechnology	Cat. # Sc-13139; RRID:AB_628445
anti-pAkt	Cell Signaling Technology	Cat # 9271S; RRID:AB_329825
anti-Akt	Cell Signaling Technology	Cat #4691; RRID:AB_915783
anti-pLIMK	Cell Signaling Technology	Cat #3841; RRID:AB_2136943
anti-pCofilin	Cell Signaling Technology	Cat #3311; RRID:AB_330238
anti-pPRAS40	Cell Signaling Technology	Cat #2997; RRID:AB_2258110
Mouse monoclonal anti-GAPDH	Millipore	Cat # MAB374; RRID:AB_2107445
Rabbit polyclonal anti-HSP90	Santa Cruz Biotechnology	Cat. # SC-7947; RRID:AB_2121236
Mouse monoclonal anti-β-tubulin	Sigma	Cat. # T8328; RRID:AB_1844090
Mouse monoclonal anti-β-actin	Sigma	Cat. # A1978; RRID:AB_476692
Goat polyclonal anti-mouse-488	Invitrogen	Cat. # A11001; RRID:AB_2534069
Goat polyclonal anti-rabbit-488	Invitrogen	Cat. # A11008; RRID:AB_143165
Goat polyclonal anti-mouse-568	Invitrogen	Cat. # A11004; RRID:AB_2534072
Goat polyclonal anti-rabbit-568	Invitrogen	Cat. # A11011; RRID:AB_143157
Alexa Fluor 568 phalloidin	Invitrogen	Cat. # A12380
Alexa Fluor 647 phalloidin	Invitrogen	Cat. # A22287; RRID:AB_2620155
Goat polyclonal anti-mouse-HRP	Dako	Cat. # P0447; RRID:AB_2617137
Goat polyclonal anti-rabbit-HRP	Dako	Cat. # P0448; RRID:AB_2617138

**Bacterial and Virus Strains**		

One Shot® TOP10 chemically competent *E. coli*	Invitrogen	Cat. # C404010
pHR’SINcPPT-SFFV (pLNT-SffV)	Vijayakumar et al., 2015	N/A
pLKO.1	Addgene; Moffat et al., 2006	Addgene Plasmid 10878
pCMVDR8.91	Vijayakumar et al., 2015	N/A
pMD.G	Vijayakumar et al., 2015	N/A

**Biological Samples**		

Human peripheral blood from healthy donors	Human peripheral blood mononuclear cells (PBMCs) were obtained from anonymised human buffy coats as supplied by the NHS Blood and Transplant (London, UK).	N/A

**Chemicals, Peptides, and Recombinant Proteins**		

Recombinant Human TGF-β1	R&D Systems	Cat. # 240-B-002
PAK4i (CRTDL4)	Cancer Research Technology	N/A
IPA-3	Santa Cruz Biotechnology	Cat. # sc-204016
Akt inhibitor		
Fibronectin from bovine plasma 0.1% solution	Sigma	Cat. # F1141
Fibronectin HiLyte 488	Cytoskeleton, Inc.	Cat. # FNR02-A
Human M-CSF	Miltenyi Biotec	Cat. # 103-093-963
MTT reagent	Sigma	Cat. # M5655
DMSO, sterile filtered	Sigma	Cat. # D2438
MCP-1 (CCL2)	R&D Systems	Cat. # 279-MC-010

**Critical Commercial Assays**		

Zero Blunt® PCR Cloning Kit	Invitrogen	Cat. # K2700-20
QuikChange® XL Site-Directed Mutagenesis Kit	Stratagene	Cat. # 200516
15ml Lymphoprep	Axis-Shield	Cat. # 1114544
CD14 Microbeads, human	Miltenyi Biotec	Cat. # 103-050-201

**Experimental Models: Cell Lines**		

THP-1 cells	ATCC	TIB-202
HEK293T cells	ATCC	CRL-3216

**Oligonucleotides**		

Cloning primer: PAK4 shRNA 2 FWD: CCGGGGTGAACATGTATG AGTGCTCGAGCACTCATACATGTTCACCTTTTTG	This paper	N/A
Cloning primer: PAK4 shRNA 2 REV: AATTCAAAAAGGTGAACATG TATGAGTGCTCGAGCACTCATACATGTTCACC	This paper	N/A
Cloning primer: PAK4 shRNA 3 FWD: CCGGCTTCGGACATTCATG ATCGCTCGAGCGATCATGAATGTCCGAAGTTTTTG	This paper	N/A
Cloning primer: PAK4 shRNA 3 REV: AATTCAAAAACTTCGGACAT TCATGATCGCTCGAGCGATCATGAATGTCCGAAG	This paper	N/A
Cloning primer: PAK4 shRNA 4 FWD: CCGGCTGCTGGACGAGTT TGAGAACCTCGAGGTTCTCAAACTCGTCCAGCAGTTTTTG	This paper	N/A
Cloning primer: PAK4 shRNA 4 REV: AATTCAAAAACTGCTGGAC GAGTTTGAGAACCTCGAGGTTCTCAAACTCGTCCAGCAG	This paper	N/A
SDM primer: PAK4 shRNA 4 rescue FWD: GCCCTCACGCTGCTC CTCGATGAGTTCGAGAACATGTC	This paper	N/A
SDM primer: PAK4 shRNA 4 rescue REV: GACATGTTCTCGAACT CATCGAGGAGCAGCGTGAGGGC	This paper	N/A
SDM primer: PAK4r(K350,351M) FWD: CTGGTGGCCGTCATGAT GATGGACCTGCGC	Wells laboratory	N/A
SDM primer: PAK4r(K350,351M) REV: GCGCAGGTCCATCATCAT GACGGCCACCAG	Wells laboratory	N/A
See Table S1 for more details of oligonucleotides used in this study		

**Recombinant DNA**		

pHR’SINcPPT-SFFV (pLNT-SffV)	Jones laboratory; Vijayakumar et al., 2015	N/A
pLKO.1	Addgene; Moffat et al., 2006	Addgene Plasmid 10878
pCMVDR8.91	Jones laboratory	N/A
pMD.G	Jones laboratory	N/A
pDEST27-PAK4	Wells laboratory	N/A
pLNT/SffV-EGFP-PAK4	This paper	N/A
pLNT/SffV-EGFP-PAK4(rescue)	This paper	N/A
pLNT/SffV-EGFP-PAK4r(K350,351M)	This paper	N/A
pLKO.1-NTC (non-targeting control shRNA)	This paper	N/A
pLKO.1-PAK4shRNA2	This paper	N/A
pLKO.1-PAK4shRNA3	This paper	N/A
pLKO.1-PAK4shRNA4	This paper	N/A

**Software and Algorithms**		

Mathematica Cell tracking notebooks	Professor Graham Dunn	N/A
DiPer	Gorelik and Gautreau, 2014	N/A
3B ImageJ plugin	Rosten et al., 2013	http://www.coxphysics.com/3b/#download
Ring protein analysis software	Staszowska et al., 2017	N/A

### Lead Contact and Materials Availability

Further information and requests for resources and reagents should be directed to and will be fulfilled by the Lead Contact, Dr Claire Wells (claire.wells@kcl.ac.uk). All unique reagents generated in this study are available from the Lead Contact with a completed Materials Transfer Agreement.

### Experimental Model and Subject Details

#### THP-1 Cells

THP-1 cells purchased from ATCC were cultured in suspension in RPMI-1640 medium (GIBCO) containing 10% heat-inactivated fetal bovine serum (FBS, Thermo Fisher Scientific), 50μM β-mercaptoethanol (Sigma) and 1% penicillin/ streptomycin (GE Healthcare). Cells were incubated at 37°C with 5% CO_2_, and maintained at a density between 5x10^5^ and 1x10^6^ cells/ml. Stocks of THP-1 cells (5x10^6^ cells/ml) were stored in 90% FBS and 10% DMSO in liquid nitrogen. Podosome formation was induced by plating cells on surfaces coated with 10μg/ml fibronectin (Sigma) in media containing 2ng/ml recombinant human TGF-β1 (R&D Systems), and incubating for 16 hours.

#### HEK293T Cells

Adherent HEK293T cells (ATCC) were cultured in RPMI-1640 medium (GIBCO) containing 10% heat-inactivated FBS, 1% penicillin/streptomycin and 2mM Glutamine (Sigma). HEK293T cells were maintained at between 50%–100% confluency by subculturing using 2ml trypsin EDTA (GE Healthcare) in PBS. Cells were maintained at 37°C with 5% CO_2_.

#### Primary Human Macrophages

Primary human monocytes were isolated from anonymous healthy donors peripheral blood samples purchased from the London Blood Transfusion service. Density gradient separation by centrifugation was carried out using 15ml Lymphoprep (Axis-Shield, Norway). Peripheral blood was diluted with PBS at a ratio of 1:2, and the cell suspension added to the density gradient before centrifuging at 800 x g at room temperature for 30 minutes. Peripheral blood mononuclear cell (PBMC) fraction was harvested and washed twice with PBS, centrifuging at 200 x g for 10 minutes at room temperature. Cells were resuspended in MACS buffer (PBS with 10% bovine serum albumin (BSA; GE Healthcare) and 0.5M EDTA), then CD14+ monocytes were isolated using magnetic bead separation using CD14 MicroBeads (Miltenyi Biotec) following the manufacturer’s protocol. To differentiate monocytes to macrophages, monocytes were seeded at a density of 0.3x10^6^ cells/ml on fibronectin coated coverslips (coated following the same protocol as for THP-1 cell differentiation). Cells were cultured in RPMI-1640 medium containing 10% FBS, 1% penicillin/streptomycin and 2mM Glutamine, in the presence of 50ng/ml M-CSF (Miltenyi Biotec) for 4.5 days.

### Method Details

#### Generation of Lentiviral Vectors

cDNA encoding wild-type human PAK4 was amplified by PCR from pDEST27-PAK4 template plasmid and subcloned into the pCR-BLUNT vector (Invitrogen; Zero Blunt® PCR Cloning Kit) while incorporating C- and N-terminal restriction sites for subsequent cloning into the pLNT/SffV lentiviral transfer vector. To generate shRNA-resistant and kinase-dead mutants, the QuikChange® XL Site-Directed Mutagenesis Kit (Stratagene) was used following the manufacturer’s instructions using the intermediate vector of PAK4 in pCR-BLUNT. Primers of approximately 20 nucleotides were designed to introduce the desired mutations. Cloned vectors were amplified using TOP10 chemically competent *E. coli* (Invitrogen).

PAK4 shRNA sequences were cloned into the lentiviral transfer vector pLKO.1 (Addgene) following the manufacturer’s protocol. Three shRNA sequences were chosen and are listed in the Key Resources Table; these sequences are numbered 2 to 4 based on previous shRNA sequences used by our laboratory. PAK4 shRNA 2 targets the same sequence as oligo 2 from Ahmed et al., 2008 in the 3′ UTR of PAK4. PAK4 shRNA 3 targets a different sequence in the 3′ UTR of PAK4, and corresponds to oligo 3 from Dart et al. (2015). PAK4 shRNA 4 targets a sequence within the coding region of PAK4 and was chosen from a list of Sigma MISSION® shRNAs, having been validated in mammalian cells.

#### Lentivirus Production

HEK293T cells were seeded at a density of 3-6x10^5^ cells/ml in 12-well plates in 1ml growth medium, and incubated at 37°C with 5% CO_2_ overnight. The following day, HEK293T cells were transfected with viral plasmids. A 500μl transfection mixture was made containing 1.3μg p8.91 packaging plasmid, 0.42μg pMD.G envelope plasmid and 1.74μg pLNT/SffV or pLKO.1 transfer plasmid and 4.35μM polyethylenimine (PEI; Invitrogen) in OptiMEM (Invitrogen). This mixture was incubated at room temperature for 15 minutes, then HEK293T cells were washed gently with OptiMEM before the transfection mix was added. Cells were then incubated at 37°C with 5% CO_2_ for 4 hours, before removing the transfection mix and adding 1ml growth medium. Transfected HEK293T cells were incubated at 37°C with 5% CO_2_ for 48 hours, before harvesting the virus by collecting the growth medium and centrifuging for 5 minutes at 2000 x g, then filtering through a 0.45μm syringe filter (Thermo Fisher Scientific).

Viral transduction of THP-1 cells was carried out by seeding 1x10^5^ THP-1 cells in 600μl growth media in each well of a 12-well plate and adding 400μl filtered lentivirus solution, with 4μg/ml polybrene (Sigma) to increase infection efficiency. Cells were incubated at 37°C with 5% CO_2_ for 72 hours before washing twice by centrifuging at 1200rpm for 5 minutes, removing media and adding 5ml PBS before centrifuging again at 1200rpm for 5 minutes. Cells were then resuspended in 3-5ml growth medium and cultured at 37°C with 5% CO_2_. For cells transduced with pLKO.1 encoding PAK4 shRNAs, cells were selected at this stage by adding 500nM puromycin (Sigma) to growth medium.

#### Inhibitor Treatment

THP-1 cells were differentiated toward a macrophage-like phenotype by seeding on fibronectin-coated coverslips in the presence of TGF-β for 16 hours. Cells were then treated with 1μM or 5μM small molecule PAK inhibitors (PAK4i from Cancer Research UK and CRUK Therapeutic Discovery Laboratories) or IPA-3 from Santa Cruz Biotechnology) or 1μM, 5μM or 10μM of Akt inhibitor (ab142088; Abcam PLC), diluted in DMSO (Sigma) and added to culture media for 4 hours while incubating at 37°C with 5% CO_2_, before being fixed in 3.7% paraformaldehyde (PFA; Sigma) in PBS for 30 minutes. See Table 1 below. For inhibitor wash-out experiments, following 4 hours incubation with inhibitors, cells were washed 3 times with fresh media and then incubated for 1-4 hours in media containing 2ng/ml TGF-β, before being fixed in 3.7% PFA in PBS. Primary human macrophages differentiated for 4.5 days with M-CSF were treated with 1μM or 5μM small molecule PAK inhibitors diluted in DMSO for 4 hours while incubating at 37°C with 5% CO_2_.

**Table:** 

Inhibitor	Source	IC_50_	Selectivity profile
PAK4i (CRTDL4)	Cancer Research Technologies	PAK1 IC_50_: 9.8 μM	The following kinases showed < 1% signal remaining after treatment with 1μM CRTDL4 in KINOME*scan* at DiscoveRx
		PAK4 IC_50_: 26.3nm	BMPR2, MEK5, PAK4, PAK6, PAK7, STK16, TGFBR2, ULK1, PSK4
IPA3	Santa Cruz Biotechnology	PAK1 IC_50_: 2.5 μM	
Akt inhibitor	Abcam PLC Catalogue number ab142088	(IC_50_ values are 58, 210 nM and 2.12 mM for Akt1, Akt2, and Akt3, respectively).	No inhibition against pleckstrin homology (PH) domain lacking Akts, PKA, PKC and SGK.

#### Podosome Counts in Fixed Cells

TGF-β differentiated THP-1 cells or M-CSF differentiated primary macrophages seeded on fibronectin coated coverslips were fixed and stained for vinculin and F-actin, and visualized using 100x objectives on LSM510 or Nikon confocal microscopes. For each coverslip, 5 distinct regions were visualized (top/bottom/left/right/center), and the number of podosomes in 20 cells per region was counted, to give a total of 100 cells per coverslip. From these counts, percentage of cells with podosomes was calculated, as well as the number of cells with 0, 1-10, 11-20, 21-30 or >30 podosomes was calculated. For each treatment condition, at least 3 coverslips were counted (> 300 cells per treatment condition) from >3 separate experiments.

#### MTT Adhesion Assay

6x10^4^ THP-1 cells per treatment condition were taken from suspension culture, centrifuged, and resuspended in 1ml fresh growth medium. Cells were pre-treated with 1μM or 5μM of the PAK inhibitors PAK4 or IPA3 diluted in DMSO, or DMSO as a control, for 1 hour while incubating at 37°C with 5% CO_2_. Cells were then taken up into 15ml falcons with 5ml PBS and centrifuged at 1200rpm for 3 minutes. Cells were resuspended in 800μl growth media containing 2ng/ml TGF-β and the same concentration of PAK inhibitor used for pre-treatment. For each treatment condition, 4 wells of a 96-well plate were coated with fibronectin as previously described, and these wells were washed twice with PBS before seeding 200μl cells per well. An additional 4 wells were coated with fibronectin and incubated with media containing TGF-β to give blank measurements. Cells were then incubated at 37°C with 5% CO_2_ for 16 hours, before dumping the plate roughly onto paper towel to remove media and unattached cells. 0.5mg/ml MTT reagent (Sigma) in PBS was sterile filtered before adding 100μl to each well and incubating at 37°C with 5% CO_2_ for 4 hours. MTT reagent was removed by needle aspiration and 100μl DMSO added to each well, pipetting up and down to mix, before incubating for a further 10 minutes at 37°C. Absorbance at 540nm was measured per well using a FLUOstar® Omega microplate reader (BMG Labtech) and blank-corrected values used. Readings from 4 wells per treatment condition x 3 separate experiments were used to give mean absorbance levels.

#### Matrix Degradation Assay

Fibronectin-488 (HiLyte Fluor 488 labeled fibronectin from bovine plasma, Cytoskeleton Inc.) coating of coverslips was carried out following the same protocol as used with unlabeled fibronectin: coverslips were inverted onto 200μl of 10μg/ml fibronectin-488 diluted in PBS, and incubated at 37°C for >3 hours. Coverslips were washed twice with PBS before seeding THP-1 cells at a density of 2x10^5^ cells/ml with 2ng/ml TGF-β for 16 hours. Cells were then fixed and stained.

Images were taken using a 100x objective on a Nikon confocal microscope and analyzed using ImageJ. Fibronectin-488 images were converted to binary images with fibronectin-488 appearing white with black degradation spots. A threshold was applied (keeping the threshold the same for all images) and the ImageJ Analyze Particles tool used to measure the total degradation area for each image. The same process was carried out using the F-actin channel to give a measure of total cell area in each image. The degradation area was divided by total cell area. For each treatment condition, >20 images were measured, and the mean degraded area/cell area (μm^2^) or degraded area (μm^2^)/cell was calculated.

#### Random Migration Assay

TGF-β differentiated THP-1 cells or M-CSF differentiated primary macrophages were seeded in fibronectin coated 96-well plates, and treated with PAK inhibitors for 4 hours. After 3 hours, 5ng/ml MCP-1 was added to induce random cell migration (1 hour prior to filming). Just before filming, 25mM HEPES (GE Healthcare) was added to each well. Cells were imaged at 37°C in phase contrast using a 10x objective on an Olympus IX-71 microscope, taking images of 2 regions per well every 2.5 minutes for 2 hours.

Cells were tracked using the ImageJ Manual Tracking tool (x/y calibration = 1.1013 pixels/μm). All cells in the starting frame were tracked, except cells that migrated outside the field of view during the duration of the film. Tracks were saved and analyzed using Mathematica notebooks developed by Professor Graham Dunn, to calculate mean cell speed for each treatment condition. To plot cell tracks, DiPer™ was used following the developers’ instructions (Gorelik and Gautreau, 2014). Approximately 30 tracks per treatment condition were plotted.

#### Western Blot and Immunoprecipitation

Adherent THP-1 cell lysates were made by seeding THP-1 cells on fibronectin-coated 10cm plates with 2ng/ml TGF-β for 16 hours, before washing twice with ice cold PBS and adding 1ml lysis buffer (4M sodium chloride, 1M Tris-HCl pH 7.4, 100mM sodium orthovanadate, 500mM sodium fluoride, 1mM EGTA, 0.5% NP-40, Roche proteinase inhibitor cocktail containing EDTA), then incubating on ice for 10 minutes. Cells were then scraped into 1.5ml Eppendorf tubes, before incubating on ice for a further 10 minutes. For non-adherent cells, THP-1 cells were counted and centrifuged at 1200rpm for 3 minutes, culture medium removed and pelleted cells washed twice with ice cold PBS before resuspending in 1ml lysis buffer and incubating on ice for 20 minutes while vortexing every 5 minutes. Lysates were then spun down at 4°C for 10 minutes at 13,000 rpm, and the supernatant was transferred to new tubes. Before running on SDS polyacrylamide gels, 5x SDS sample buffer was added to lysates before heating at 95°C for 5 minutes. SDS-PAGE was carried out using 7.5%–12% gels and transferring onto nitrocellulose membranes. To immunoblot for protein levels, membranes were blocked with 5% milk in TTBS before probing with primary antibodies followed by secondary antibodies listed in the Key Resources Table. Membranes were immersed in in enhanced chemiluminescence reagent (ECL, Amersham) for 1 minute before imaging using a Bio-Rad ChemiDoc MP imaging system.

For immunoprecipitation, 6x10^7^ TGF-β differentiated THP-1 cells were lysed in 1ml lysis buffer. 30μl lysate with 20μl 5x SDS sample buffer was kept at −20°C as input. The remaining lysate was incubated with 1-2μg antibody for immunoprecipitation overnight at 4°C while rotating. 60μl protein A/G agarose beads were washed 3 x in lysis buffer, and lysate/antibody samples added to the beads, before rotating at 4°C for 1 hour. Samples were centrifuged at 4000rpm for 3 minutes at 4°C and the supernatant removed, before washing beads 3x by incubating with 1ml lysis buffer for 5 minutes while rotating at 4°C. Lysis buffer was removed before adding 30μl 5x SDS sample buffer and boiling for 10 minutes to precipitate proteins from the beads. Immunoprecipitates were loaded on polyacrylamide gels and western blots carried out.

#### Immunocytochemistry

Cells were stained for immunofluorescence imaging at room temperature as follows: cells were fixed with 3.7% PFA for 30 minutes, then washed 3x with PBS before permeabilization with 0.1% Triton X-100 (Sigma) in PBS for 5 minutes. Cells were again washed 3x with PBS, then blocked with 3% BSA in PBS for 30 minutes, before incubating with primary antibody diluted in 3% BSA in PBS for 1 hour. Cells were then washed 4x for 5 minutes per wash with PBS, before incubating with secondary antibody diluted in 3% BSA in PBS for 30 minutes. Cells were again washed 4x for 5 minutes per wash with PBS, before mounting coverslips. Coverslips were washed with ddH_2_0 before mounting on glass slides on 15μl mounting media (MOWIOL, Sigma) and incubating at 37°C for 20 minutes before storing at room temperature in the dark. For all antibodies used, see the Key Resources Table.

#### Imaging

Images of fixed cells were acquired using 100x oil Plan Fluor immersion objectives (numerical aperture 1.4) on Zeiss LSM510, Nikon TIRF or Nikon Spectral confocal microscopes. Excitation wavelengths of 488nm, 543nm and 633nm were used. Images were converted to PNG files from database files.mdb or ND2 files using ImageJ, and merged or single channel images generated using ImageJ. For live cell imaging, a 10x objective on an Olympus IX-71 wide-field inverted microscope equipped with a 37°C environment chamber was used. Image acquisition was controlled by Metamorph imaging software (Universal Imaging), and images were taken in phase contrast every 2.5 minutes for 2 hours. Combined STORM and 3B imaging was carried out with Nikon N-STORM microscope. For STORM imaging, 10,000 frames per sample were processed with ThunderSTORM software (Ovesný et al., 2014) using default processing parameters. For 3B analysis, datasets of >200 images were taken using stream acquisition. A 100x oil immersion objective on an Olympus IX-81 wide-field inverted microscope was used, and imaging controlled by Metamorph imaging software. 3B analysis was carried out using the ImageJ 3B plugin, following instructions in Rosten et al. (2013). For datasets where two channels are analyzed, the exact same selection region was applied to both channels using coordinates listed by ImageJ. Analysis of ring protein localizations was carried out as described in Staszowska et al. (2017).

### Quantification and Statistical Analysis

Focal adhesions were quantified using ImageJ as follows: vinculin images were converted to binary images, a threshold applied and the ImageJ Analyze Particles tool used to measure the mean number of adhesions per cell. Statistical analyses were performed using SPSS Statistics software (IBM). Checks for normality were performed before the appropriate statistical test for the data was chosen. Student’s t test or one-way analysis of variance (ANOVA) was used; for each set of data, the test used and n values are indicated in the figure legend. Results were defined as significantly different with a p value of < 0.05.

### Data Code and Availability

This study did not generate any unique datasets or code. All data are contained within the paper.

## Supplemental Information

Document S1. Figures S1–S4 and Table S1

Document S2. Article plus Supplemental Information
