# PAK4 Kinase Activity Plays a Crucial Role in the Podosome Ring of Myeloid Cells

**DOI:** 10.1016/j.celrep.2020.107593

**Published:** 2020-04-21

**Authors:** Elizabeth Foxall, Adela Staszowska, Liisa M. Hirvonen, Mirella Georgouli, Mariacristina Ciccioli, Alexander Rimmer, Lynn Williams, Yolanda Calle, Victoria Sanz-Moreno, Susan Cox, Gareth E. Jones, Claire M. Wells

(Cell Reports *29*, 3385–3393.e1–e6, December 10, 2019)

In the originally published version of this article, the author name Victoria Sanz Moreno was spelled incorrectly and should be Victoria Sanz-Moreno. This has now been corrected online.

The authors regret this error.

