# Supplementary material for: PAK4 Kinase Activity Plays a Crucial Role in the Podosome Ring of Myeloid Cells
Source: Cell Rep. 2019 Dec 10;29(11):3385–3393.e6. doi: 10.1016/j.celrep.2019.11.016 (PMC6915307; doi:10.1016/j.celrep.2019.11.016)
Supplement: Document S1. Figures S1–S4 and Table S1 [file mmc1.pdf]

Cell Reports, Volume *29*

Supplemental Information

PAK4 Kinase Activity Plays a Crucial Role

in the Podosome Ring of Myeloid Cells

Elizabeth Foxall, Adela Staszowska, Liisa M. Hirvonen, Mirella Georgouli, Mariacristina Ciccioli, Alexander Rimmer, Lynn Williams, Yolanda Calle, Victoria Sanz Moreno, Susan Cox, Gareth E. Jones, and Claire M. Wells

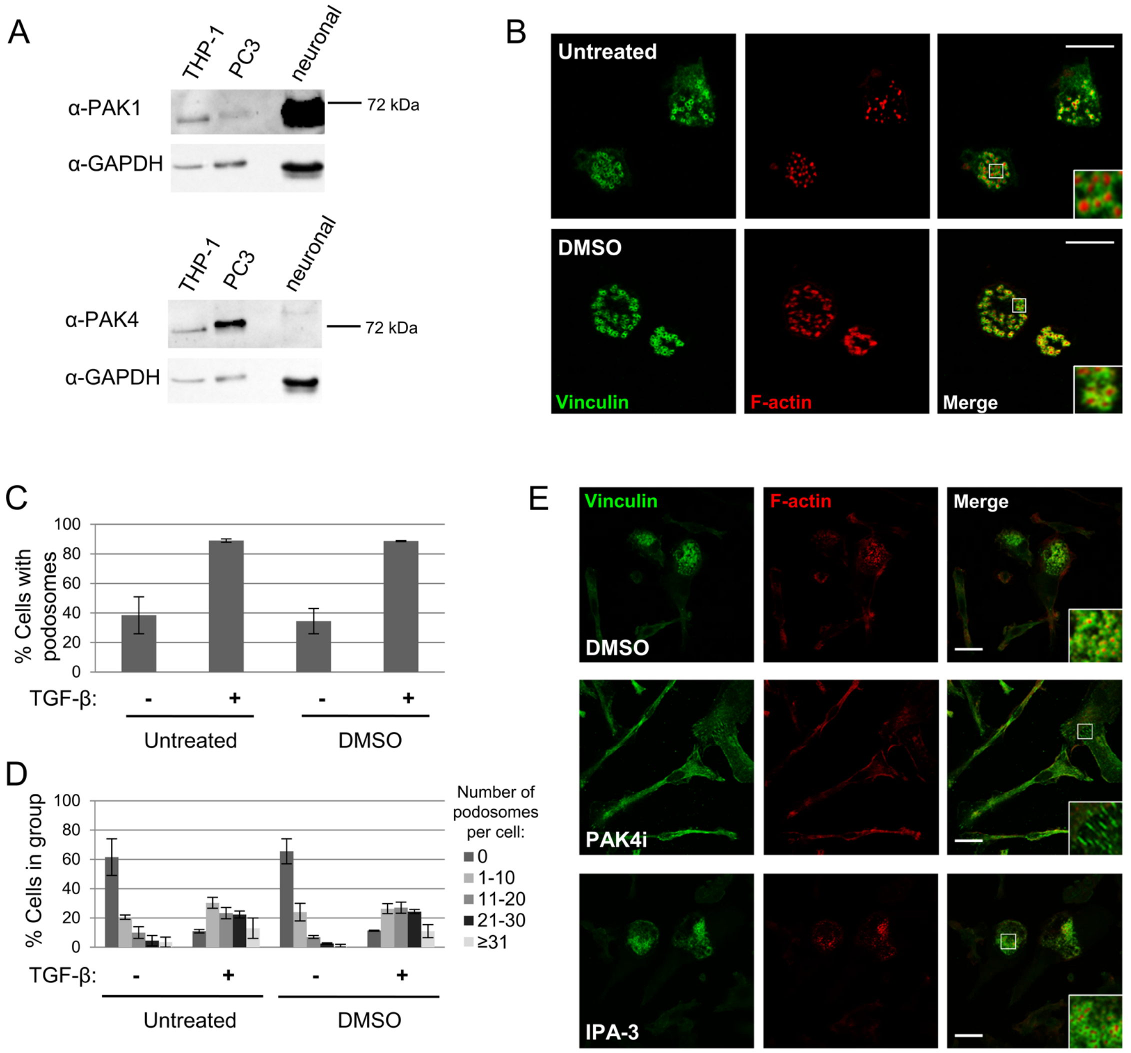

Figure S1. PAK1 and PAK4 inhibition suppresses podosomes. Related to Figure 1 (A) Western blots for PAK1 and PAK4 expression levels in THP-1 cells, tested alongside prostate cancer and neuronal cell lysates. (B) THP-1 cells seeded on fibronectin with TGF-β for 16 hours were left untreated or treated with DMSO for 4 hours before being fixed and stained for vinculin (green) and F-actin (red). (C) THP-1 cells from suspension (-TGF-β) or THP-1 cells seeded on fibronectin with TGF-β for 16 hours (+TGF-β) were left untreated or treated with DMSO for 4 hours, and the percentage of cells with podosomes and (D) the percentage of cells with 0, 1-10, 11-20, 21-30 or ≥31 podosomes per cell was calculated from >300 cells per condition. Error bars = +/- SEM. Confocal images of (E) THP-1 cells or (F) primary macrophages treated with 5μM PAK inhibitors for 4 hours before fixing and staining for vinculin (green) and F-actin (red). All scale bars = 10μm.

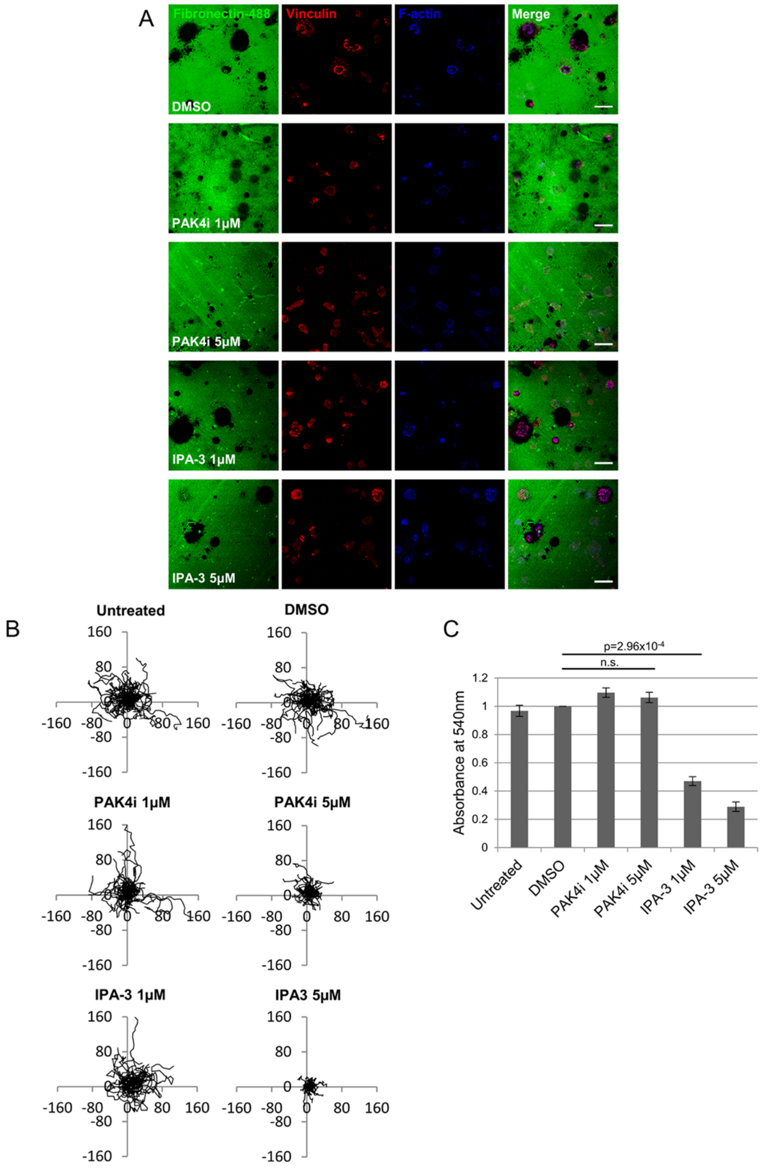

Figure S2. Inhibition of PAK1 and PAK4 reduces migration and adhesion. Related to Figure 2 (A) Confocal images of THP-1 cells seeded on fibronectin-488 with TGF-β for 16 hours before treating with PAK inhibitors for 4 hours. (B) THP-1 cells seeded on fibronectin with TGF-β for 16 hours were stimulated 1 hour prior to filming with MCP-1, and images taken every 2.5 minutes for 2 hours in phase contrast. Cells were then tracked using the ImageJ Manual Tracking tool, and cell tracks were plotted using DiPerTM [38]. Axes = µm from starting point 0. Approximately 30 cell tracks are plotted per treatment condition. (C) THP-1 cells in suspension were pre-treated with PAK inhibitors for 1 hour, before seeding on fibronectin with TGF-β for 16 hours, in the presence of PAK inhibitors. Media was dumped from the wells by inverting the plate, then MTT reagent was added to the wells, and cells incubated at 37°C for a further 4 hours. MTT in PBS was removed by needle aspiration, and DMSO added to each well, before incubating at 37°C for 10 minutes. Absorbance at 540nm was then read using a microplate reader. IPA-3 5µM treatment is not included, since these cells did not adhere. Error bars = +/- SEM and p values denote significant difference to control DMSO treated cells by one-way ANOVA.

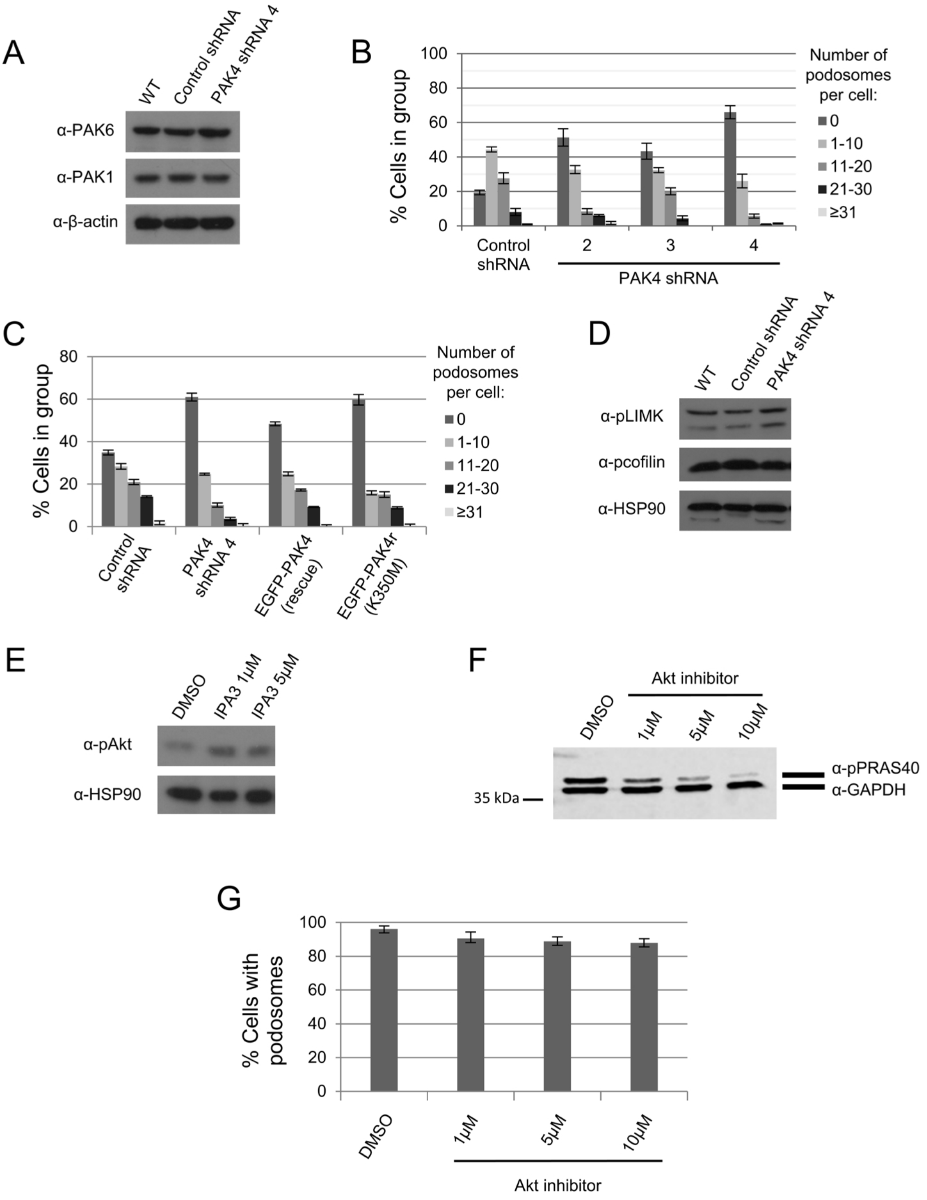

Figure S3. PAK4 knockdown supports a kinase-dependent role in podosomes, via activation of Akt. Related to Figure 3 (A) Parental THP-1 cells, or cells stably expressing PAK4 shRNA 4 or a scrambled control were lysed and immunoblotted for expression of PAK1 and PAK6. (B) PAK4 shRNA expressing cells or (C) PAK4 rescue cells were seeded on fibronectin with TGF-β for 16 hours before fixing and staining for vinculin and F-actin. The percentage of cells with 0, 1-10, 11-20, 21-30 or ≥31 podosomes per cell was calculated from 300 cells per condition. (D) Lysates from parental THP-1 cells and cells expressing control shRNA or PAK4 shRNA 4 were probed for pLIMK and pcofilin. (E) THP-1 cells were seeded on fibronectin with TGF-β for 16 hours before treating with 1μM or 5μM IPA3 for 4 hours. Cell lysates were probed for pAkt. (F) THP-1 cells on fibronectin with TGF-β for 16 hours were treated with indicated concentrations of Akt inhibitor for 4 hours. Cell lysates were probed for inhibition of Akt signalling (anti-PRAS40) and GAPDH as a loading control. (G) THP-1 cells on fibronectin with TGF-β for 16 hours were treated with indicated concentrations of Akt inhibitor for 4 hours. The inhibitor was then washed out and the cells incubated for a further 12 hours. Cells were fixed and stained for F-actin. Cells were scored for the presence of podosomes. Error bars = +/- SEM.

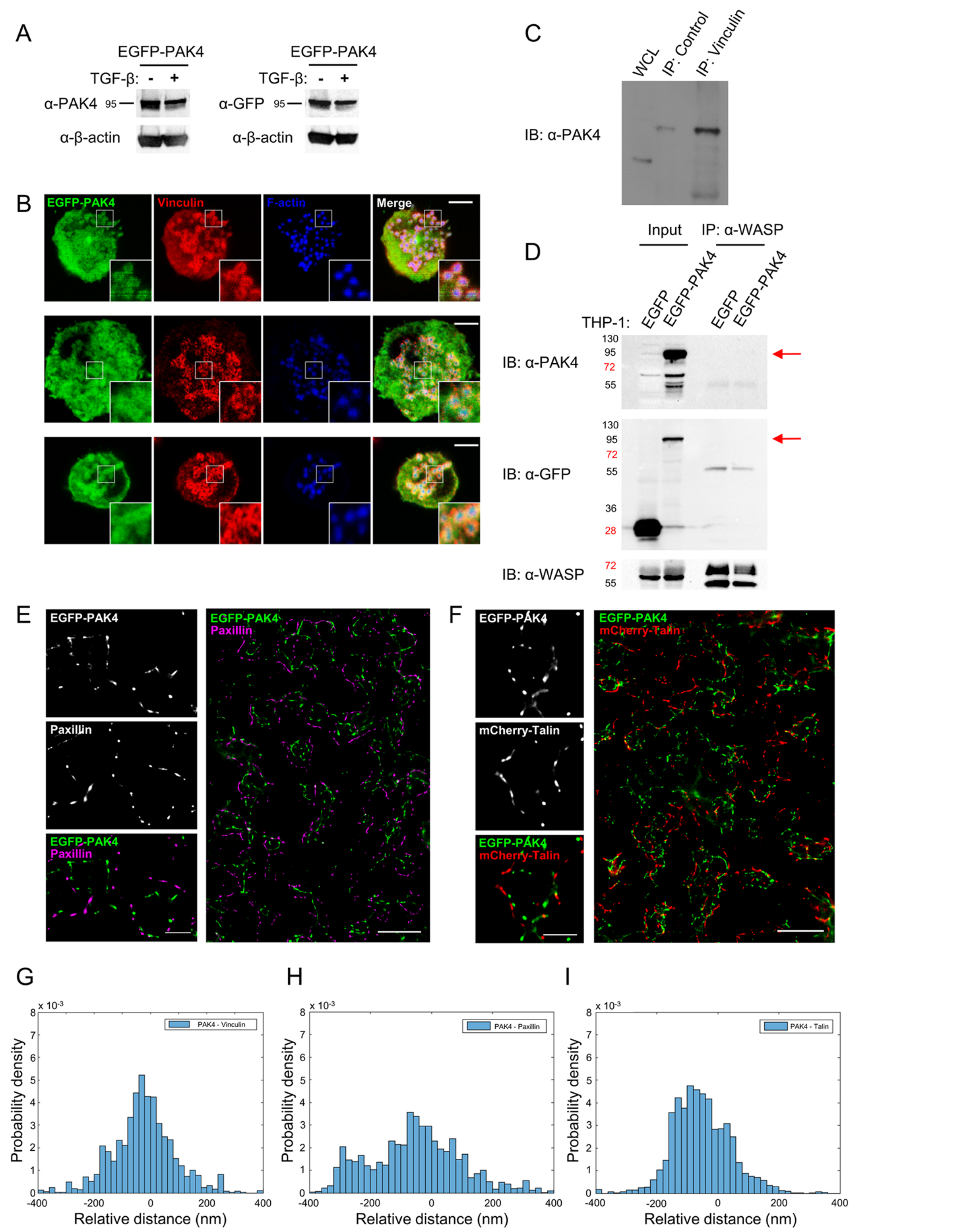

Figure S4. 3B analysis of PAK4 localisation. Related to Figure 4 (A) THP-1 cells stably expressing EGFP-PAK4 were generated by lentiviral infection. Lysates were collected from suspension culture (-TGF-β) as well as cells following TGF-β mediated differentiation (+TGF-β), western blot for PAK4 expression levels (left) or anti-GFP(right). Blots shown are representative of three separate experiments. (B)EGFP-PAK4 expressing THP-1 cells were seeded on fibronectin with TGF-β for 16 hours before fixing and staining for vinculin (red) and F-actin (blue). Scale bar =5μm. (C) THP-1 cells stably expressing EGFP-PAK4 were seeded on fibronectin with TGF-β for 16 hours before harvesting cell lysates. Immunoprecipitation (IP) of vinculin was carried out before immunoblotting (IB) for PAK4. (D) THP-1 cells stably expressing EGFP or EGFP-PAK4 were seeded on fibronectin with TGF-β for 16 hours before harvesting cell lysates. IP of WASP was carried out before IB for PAK4; blots were stripped and reprobed for GFP followed by WASP. (E) EGFP-PAK4 expressing THP-1 cells were seeded on fibronectin with TGF-β for 16 hours before fixing and staining for paxillin. and (F) EGFP-PAK4/mCherry Talin expressing cells were seeded on fibronectin with TGF-β for 16 hours. In E and F bar in left hand panel = 500nm and bar in right hand panel = 2 μm. Datasets were taken as indicated in Figure 4 (G,H and I) Histograms show the relative positions of EGFP-PAK4 and vinculin/paxillin/mCherry-Talin in the podosome ring calculated by subtracting ring position distance from PAK4 distance. Negative values indicate that PAK4 is closer to the podosome centre.

Cloning primer: PAK4 BamHI FWD: CGCGGATCCGGTGGAGGAATGTTTGGGAAGAGGAAGAAGC	This paper	N/A
Cloning primer: PAK4 XhoI REV: CCGCTCGAGTCATCTGGTGCGGTTCTG	This paper	N/A
Sequencing primer: GFP end FWD: GGCATGGACGAGCTGT	Jones laboratory	N/A
Sequencing primer: PAK4 internal FWD: CGGCCCTTTAACACCTACCCGA	Jones laboratory	N/A
Sequencing primer: PAK4 internal REV: CGTTCATCCTGGTGTGGGTGACGA	Jones laboratory	N/A
Sequencing primer: PAK4 internal REV 2: CCGTGAACTTCTGCTCGTG	This paper	N/A
Sequencing primer: pLNT/SffV 5' FWD: ACCTGAAATGACCCTGCG	Jones laboratory	N/A
Sequencing primer: pLNT/SffV 3' REV: CATGCCTGCAGGTCGACTCTA	Jones laboratory	N/A
Sequencing primer: pLKO.1 FWD: CAAGGCTGTTAGAGAGATAATTGGA	Addgene	N/A

Table S1. Related to STAR METHODS. Additional information on oligonucleotides used in this study
